# Severe Adverse Drug Reactions to Quetiapine in Two Patients Carrying CYP2D6*4 Variants: A Case Report

**DOI:** 10.3390/ijms22126480

**Published:** 2021-06-17

**Authors:** Céline K. Stäuble, Markus L. Lampert, Thorsten Mikoteit, Martin Hatzinger, Kurt E. Hersberger, Henriette E. Meyer zu Schwabedissen

**Affiliations:** 1 Biopharmacy, Department of Pharmaceutical Sciences, University of Basel, 4056 Basel, Switzerland; h.meyerzuschwabedissen@unibas.ch; 2 Pharmaceutical Care, Department of Pharmaceutical Sciences, University of Basel, 4001 Basel, Switzerland; markus.lampert@unibas.ch (M.L.L.); kurt.hersberger@unibas.ch (K.E.H.); 3 Institute of Hospital Pharmacy, Solothurner Spitäler, 4600 Olten, Switzerland; 4 Psychiatric Services Solothurn, Solothurner Spitäler and Department of Medicine, University of Basel, 4503 Solothurn, Switzerland; thorsten.mikoteit@spital.so.ch (T.M.); martin.hatzinger@spital.so.ch (M.H.)

**Keywords:** pharmacogenetics, pharmaceutical care, psychiatry, depression, neuroleptics, antipsychotics, quetiapine, CYP2D6, CYP3A4, adverse drug reaction

## Abstract

We report two cases of patients who developed severe adverse drug reactions including persistent movement disorders, nausea, and vertigo during treatment with quetiapine at maximum daily doses ranging between 300 and 400 mg. The extensive hepatic metabolism of quetiapine is mainly attributed to cytochrome P450 3A4 (CYP3A4). However, there is recent evidence supporting the idea of CYP2D6 playing a role in the clearance of the quetiapine active metabolite norquetiapine. Interestingly, both patients we are reporting of are carriers of the CYP2D6*4 variant, predicting an intermediate metabolizer phenotype. Additionally, co-medication with a known CYP2D6 inhibitor and renal impairment might have further affected quetiapine pharmacokinetics. The herein reported cases could spark a discussion on the potential impact of a patient’s pharmacogenetic predisposition in the treatment with quetiapine. However, further studies are warranted to promote the adoption of pharmacogenetic testing for the prevention of drug-induced toxicities associated with quetiapine.

## 1. Background

Patients suffering from major psychiatric disorders often need long-term pharmacotherapy in order to reach remission and prevent relapse. Considering that, it seems even more important to select and prescribe safe and well-tolerated pharmacotherapies. However, interindividual variability in response to psychotropic drugs is well known, and adverse drug reactions (ADRs) are common. In fact, severe ADRs, requiring or prolonging hospitalization or limiting self-care and activities of daily life, are on average reported for 1–2% of psychiatric inpatients under treatment with psychotropic drugs [1,2,3]. In particular, excessive systemic drug exposure may increase the risk of experiencing unwanted side effects and toxicity. Apart from dosing errors, increased systemic drug exposure can also occur under regular dosing and may be attributed to drug–drug or food–drug interactions, impaired renal or hepatic elimination, and notably, individual genetic predisposition. The latter is of relevance when polymorphisms affect the expression and/or activity of genes encoding enzymes and transporters involved in drug absorption, distribution, metabolism, or excretion (ADME). As an illustration, cytochrome P450 2D6 (CYP2D6), which is highly polymorphic, has several known genetic variants translating into increased, reduced, or even lacking enzyme activity. Accordingly, phenotypes are termed ultrarapid, intermediate, or poor metabolizers, respectively. Systemic exposure of active drug molecules that are extensively metabolized by CYP2D6 may be elevated in individuals carrying genetic variants translated into CYP2D6 enzymes with reduced or no activity [4]. In the case of several antipsychotics, namely, aripiprazole, haloperidol, risperidone, and zuclopenthixol, a gene–drug interaction with CYP2D6 has been rated as actionable, meaning that there is clinical evidence for dose adaptation to the respective geno- or phenotype. Accordingly, to prevent toxicities, a dose reduction is recommended for patients with predicted reduced CYP2D6 activity [5], and drug labels draw attention to possible risks [6]. Currently, no such pharmacogenetic recommendations are available for the widely prescribed atypical antipsychotic quetiapine.

**Figure 1:**
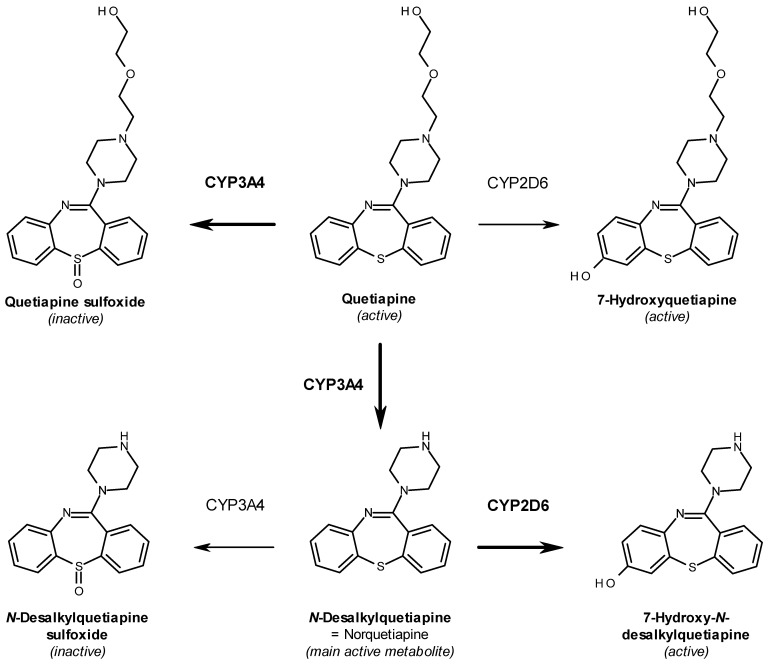
Illustration of major steps in phase I metabolism of quetiapine.

Quetiapine is indicated and approved for the treatment of schizophrenia and bipolar disorder but also as a supplementary treatment for depressive episodes in patients inadequately responding to antidepressant monotherapy [7]. It is known that, in contrast to typical antipsychotics, quetiapine and its main active metabolite norquetiapine show increased selectivity for the serotonin receptor 2A (*HTR2A*) over the dopamine receptor (*DRD2*) [8,9] and are therefore associated with a limited risk of extrapyramidal symptoms [10]. Quetiapine exhibits an antagonistic mechanism of action at the aforementioned receptors, which is assumed to be responsible for its antipsychotic effect [11]. Moreover, quetiapine is also effective as an augmentation in the treatment of depressive episodes [7], which is attributed to its active metabolite norquetiapine and its high affinity for both the noradrenaline transporter (*SLC6A2*) and the serotonin receptor 1A (*HTR1A*), towards which it was shown to exhibit inhibitory and partial-agonistic activity, respectively [12,13]. Apart from the abovementioned targets, responsible for the therapeutic effect of quetiapine, there are also several known off-target interactions assumed to be linked to some of the frequently reported side effects in the treatment with quetiapine [14]. Both quetiapine and norquetiapine, for example, show relevant affinity for and antagonistic activity upon binding to the histaminergic (*HRH1*) and the adrenergic alpha 1 (*ADRA1*) receptors [12], which may cause symptoms like sedation and hypotension [7]. Furthermore, norquetiapine also binds to muscarinic (*CHRM1*) receptors [12], which may cause the often-observed anticholinergic side effects including dry mouth, constipation, and tachycardia [7]. Following absorption, quetiapine undergoes extensive hepatic metabolism mainly catalyzed by CYP3A4, which, inter alia via *N*-dealkylation, gives rise to the main active metabolite norquetiapine (*N*-desalkyl-quetiapine) (Figure 1) [15]. Even if less than 5% of the unaltered mother substance is renally eliminated, over 70% of quetiapine metabolites are excreted via urine [7]. Currently, dose adaptation is not recommended for renally impaired patients [7].

## 2. Case Presentation

We herein report two cases of severe ADRs related to quetiapine in patients who received a pharmacogenetic consultation by clinical pharmacists. Currently, this consultation is part of an observational study approved by the local ethics committee (EKNZ ID: 2019-01452), and the patients’ consent was obtained prior to the intervention (ClinicalTrials.gov identifier: NCT04154553). Panel-pharmacogenotyping was conducted by using the commercial service Stratipharm^®^ offered by humatrix AG (Pfungstadt, Germany). In their laboratory, polymorphisms are determined by applying real-time PCR using the automated Life Technologies QuantStudio 12 k flex (Thermo Fisher, Waltham, MA, USA) with the respective optimized and commercially available chemistry.

### 2.1. Case #1: Movement Disorder and Constipation

A 63-year-old male patient with a history of bipolar affective disorder (ICD-10 F31) type II, was admitted to our clinic for inpatient treatment due to an acute worsening of a depressive episode. Herein, he was diagnosed with a currently moderate depressive episode (ICD-10 F31.3) quantified by a rater-assessed 21-item Hamilton Rating Scale of Depression (HAM-D21) [16] with a score of 27 and a patient-assessed Beck Depression Inventory (BDI) [17] with a score of 31. Prior out-patient treatment attempts of the current depressive episode with trazodone 150 mg daily and later agomelatine 50 mg daily were ineffective. Furthermore, due to a previously diagnosed hypertensive and arrhythmogenic cardiomyopathy with an implanted cardiac pacemaker and a history of venous thrombosis, he was under co-medication with rivaroxaban (20 mg/d), eplerenon (25 mg/d), azilsartan (40 mg/d), and chlorthalidone (12.5 mg/d) (Table 1). Additionally, a history of congenital ureteral stenosis and thereafter unilateral nephrectomy caused a chronic renal insufficiency currently staged G3a, with measured eGFR CKD-EPI between 46 and 55 mL/min/1.73 m^2^. As stated above, the goal of the current hospitalization was the adjustment of medications in order to treat the bipolar disorder currently presenting a moderate bipolar II depression. Therefore, vortioxetine was added to the already installed agomelatine and dosed up to 20 mg daily. Concomitantly, a treatment with quetiapine was started as first-line medication for bipolar depression and as an augmentation to the antidepressant treatment. For an optimal effect, the administration of quetiapine was split into two doses, an extended-release (XR) evening dose and a non-retarded night dose. Herein, quetiapine dosage was gradually increased over the course of three weeks to cumulative 400 mg daily (Table 1). Upon reaching this maximum dosage, the patient suddenly showed a strong sedation and severe movement disorders, which manifested as a persistent tremor. At the same time, the patient also complained of severe constipation. Thus, a laxative was prescribed, and quetiapine dosage was again reduced to 100–200 mg daily, which was well tolerated by the patient and led to remission of the aforementioned side effects. However, after one month in the clinic, the patient still showed no significant clinical improvement in depression. Therefore, the antidepressant treatment was again changed from vortioxetine to bupropion with a well-tolerated maximum dosage of 300 mg. Moreover, the patient was simultaneously referred to a consultation by clinical pharmacists of the hospital for an in-depth medication review including pharmacogenetic testing and counselling. Interpretation of the genotyping results identified the patient as a CYP2D6 intermediate metabolizer (IM, *4 heterozygous), CYP2C19 intermediate metabolizer (IM, *2 heterozygous), and CYP2B6 wildtype (WT, *1 homozygous) phenotype. Furthermore, the patient showed genetic variants resulting in increased inducibility of CYP1A2 (*1F homozygous) and no variation in the *ABCB1* polymorphism rs2032583 (Table 2). Based on these results, the switch to bupropion was considered appropriate, and no further antidepressant medication change was recommended. Indeed, the patient could finally be discharged after 9 weeks of hospitalization under remission, quantified by a HAM-D21 score of 6 and a BDI score of 11.

### 2.2. Case #2: Emesis and Vertigo

A 26-year-old male was admitted to our clinic after a suicide attempt. Due to untreated, pre-existing arterial hypertension and tachycardia (diastolic pressure >100 mmHg and heart rate >100 bpm) at clinic entry, first of all, a treatment with lisinopril 7.5 mg daily was prescribed. In the further course of hospitalization, the patient was diagnosed with a moderate depressive episode (ICD-10 F32.1) based on clinical symptoms, predominantly sadness, anhedonia, amotivation, anxiety, pessimism, and insomnia. Subsequently, an antidepressant treatment with escitalopram 10 mg daily was initiated, with good tolerance. Meanwhile, due to pronounced circling thoughts and tension, an additive treatment with quetiapine at 50 mg daily was started. In the fourth week of hospitalization, the patient showed continuous tachycardia (heart rate >100 bpm), whereupon treatment with metoprolol 25 mg daily was started, and the dosage of the already established lisinopril was increased to 10 mg daily. At the same time, due to persistent sleeping disorder and circling thoughts, the dosage of quetiapine was increased to cumulative 300 mg daily over a period of 5 days (Table 3). Upon reaching the maximum quetiapine dosage, the patient suddenly developed massive and continuous emesis and vertigo with an unsteady gait. Due to lack of recovery after two days, the patient was transferred to the medical department for further evaluation. After cardiological and neurological assessment, the patient was diagnosed with a postural orthostatic tachycardia syndrome (normotonic, heart rate > 100 bpm). As a first intervention, quetiapine was slowly reduced and finally discontinued. Furthermore, as advised by internists and neurologists, lisinopril was stopped as well, and metoprolol dosage was increased to 75 mg, administered in two doses. Thereby, the aforementioned severe ADRs remitted. In the further course, escitalopram dosage was increased to 20 mg daily, and low-dose trazodone 100 mg daily plus pregabalin up to 150 mg daily, indicated for anxiety-related sleep-onset insomnia, were successfully established. Thus, the sleeping disorder and the depression improved markedly. Meanwhile, due to the aforementioned severe side effects, the patient was referred to a consultation by clinical pharmacists of the hospital for an in-depth medication review including pharmacogenetic testing and counselling. Interpretation of the genotyping results identified the patient as a CYP2D6 intermediate metabolizer (IM, *4 heterozygous), CYP2C19 intermediate metabolizer (IM, *2 heterozygous), and CYP2B6 wildtype (WT, *1 homozygous) phenotype. Furthermore, the patient showed genetic variants resulting in increased inducibility of CYP1A2 (*1F heterozygous), and no variation in the *ABCB1* polymorphism rs2032583 (Table 4). Based on these results and the continuous clinical improvement of the patient, no further adjustments of the medication were necessary, and the patient was discharged in a stabilized condition after 12 weeks of inpatient treatment. 

## 3. Discussion and Conclusions

We report on two patients experiencing pronounced adverse drug reactions. In the first case, the patient showed a sudden onset of severe movement disorders and constipation after increasing the quetiapine daily dose to 400 mg. In a second case, the patient developed persistent nausea and vertigo, diagnosed as a postural orthostatic tachycardia syndrome, when the daily dosage of quetiapine was increased to 300 mg. All of the aforementioned side effects are observed frequently (1–10%) to very frequently (>10%) in patients treated with quetiapine [7]. Due to the temporal relationship between the onset of strong symptoms and the increase of quetiapine dosage, an excessive, systemic exposure to quetiapine could be suspected. However, in both cases, quetiapine blood concentrations were not measured as part of the clinical routine. Rather, the treating physicians attempted a quetiapine dose reduction, which led to remission of the afore-described side effects in both cases and, as a result, may further support the hypothesis of dose-dependent induced adverse reactions to quetiapine. A closer look at the pharmacogenetic profiles revealed that both patients carry a CYP2D6*4 variant, most likely translating into an enzyme with reduced activity and giving rise to the so-called intermediate metabolizer phenotype. Even if there are no recommendations on quetiapine use or dosing in patients genotyped for CYP2D6, we want to highlight that there are data supporting a role for this enzyme in quetiapine metabolism alongside with CYP3A4. More precisely, CYP2D6 was found to catalyze the 7′-hydroxylation of quetiapine and its active metabolite norquetiapine, leading to the formation of active metabolites, namely, 7-hydroxyquetiapine and 7-hydroxy-N-desalkylquetiapine (Figure 1) [18,19]. However, 7′-hydroxylation via CYP2D6 might be an important route of clearance for the main active metabolite norquetiapine, as in vitro data showed a significantly higher affinity for CYP2D6 compared to CYP3A4 (Figure 1) [19]. This is further supported by clinical data showing that the intake of strong CYP3A4 inductors influences quetiapine but exhibits only a limited effect on norquetiapine serum concentration [20]. Moreover, *CYP2D6* polymorphisms with predicted reduced activity have been associated with increased norquetiapine serum concentrations by 22 and 30% for intermediate and poor metabolizers, respectively, compared to normal metabolizers [21]. It seems noteworthy in this context that clinical data showed serum concentrations of norquetiapine at steady state to be almost two-fold higher compared to those of quetiapine [20]. In addition, the elimination half-life of norquetiapine was reported to be of 12 h, which is notably longer compared to 7-h half-life reported for the mother substance quetiapine [7]. Distinct differences between quetiapine and norquetiapine can also be found in their pharmacologic profiles. Apart from the postulated norquetiapine antidepressant activity via interaction with the noradrenaline transporter (*SLC6A2*) and the serotonin receptor 1A (*5HTR1A*), a remarkably higher affinity for the histamine H1 (*HRH1*) and muscarinic M1 (*CHRM1*) receptors was detected, compared to quetiapine [12]. These histaminergic and muscarinic off-target effects may be associated with some of the known side effects under treatment with quetiapine [7,12] and may also be associated with the observed side effects in the herein reported cases, including drowsiness, nausea, sedation, constipation, and tachycardia. 

We further found that, in both cases, additional factors might have influenced quetiapine clearance. In the first case, the patient exhibited a relevant renal impairment, which may have further slowed down drug clearance, as over 70% of the partly active quetiapine metabolites are excreted renally [7]. However, quetiapine dosage reduction is currently not recommended for renally impaired patients, and studies on the topic are sparse. It may be speculated that reduced CYP2D6 activity and renal impairment may have had an additive effect on the overall clearance of quetiapine and its metabolites. In the second case, the patient was co-medicated with escitalopram, a known CYP2D6 inhibitor [22,23]. Due to his genetic predisposition, with an already reduced CYP2D6 activity, this might have additively affected quetiapine clearance. Phenoconversion is the deviation from an individual’s genotype-predicted phenotype and is caused by nongenetic factors such as comedication, comorbidities, or nutrition [24]. It is suspected that, especially in the case of genetic intermediate metabolizers, the addition of an enzyme inhibitor may lead to the phenotypic display of an actual poor metabolizer [24]. In the first case, switching to the known CYP2D6 inhibitor bupropion [25] was, however, well tolerated in combination with quetiapine at the already lowered dosage of 100–200 mg daily. This may point out the importance of pre-emptive measures such as dose reduction to support the prescription of safe and efficient therapies in cases like these. For the second case, we want to mention that it should certainly be realized that the reported ADRs may also be linked to the antihypertensive medication initiated at hospitalization. Indeed, side effects including nausea and vertigo are also reported for metoprolol and lisinopril. Additionally, metoprolol clearance may as well be affected by alterations in CYP2D6 activity [7]. However, after remission of the reported ADRs, the patient well tolerated an increase of metoprolol dosage from 25 to 75 mg daily. Still, the reported, pronounced adverse effects may be conclusively linked to quetiapine, taking into account additive factors, such as genetic predisposition, comedication, and renal function, likely affecting its pharmacokinetics.

At present, the impact of CYP2D6 and its genetic variants on overall quetiapine and, especially, norquetiapine clearance is still not well elucidated, and further research is needed to allow a recommendation for its management in clinical practice. On the one hand, cases like these, including our recently reported cases on antidepressants and tamoxifen [26,27], point out the complexity and the yet still fragmentary available evidence, making the integration of pharmacogenetic data into clinical practice challenging. On the other hand, the consideration of pharmacogenetic predispositions may offer additional insights for a better understanding of adverse drug reactions as well as of non-response and create an opportunity for healthcare professionals to further enhance safety and effectiveness of marketed drugs. 

## Figures and Tables

**Table 1:** Case #1 medication at the time of the reported severe ADRs.

Substance	Schedule
Quetiapine XR ^1^ 200 mg	0-0-1-0
Quetiapine 200 mg	0-0-0-1
Agomelatin 25 mg	0-0-0-2
Vortioxetine 10 mg	2-0-0-0
Lactitol 667 mg/mL	20-0-0-20 mL
Rivaroxaban 20 mg	1-0-0-0
Eplerenon 25 mg	1-0-0-0
Azilsartan/Chlorthalidone 20/12.5 mg	1-0-0-0

^1^ XR: extended release.

**Table 2:** Case #1 selected results of the panel-pharmacogenotyping and phenotype interpretation.

Gene	Variant *(Also Tested Variants in Gene Locus)*	Genotype	Predicted Phenotype
*CYP1A2*	rs762551 g.75041917C > A (in *1F) *(rs2069514)*	A/A	Increased inducibility
*CYP2B6*	*(rs8192709*, *rs28399499*, *rs3745274)*	WT ^4^, *1	Normal function (NM ^1^)
*CYP2C19*	rs4244285 c.681G > A (in *2) *(rs4986893*, *rs12248560, rs28399504)*	G/A	Decreased function (IM ^2^)
*CYP2D6*	rs3892097 c.506-1G > A (in *4)	G/A C/T	Decreased function (IM ^2^)
	rs1065852 c.100C > T (in *4) *(CNV, rs35742686*, *rs5030655*, *rs5030867*, *rs5030865*, *rs5030656*, *rs201377835*, *rs28371706*, *rs59421388*, *rs28371725)*		
*CYP3A4*	rs2242480 c.1026+12G > A (in *1B) *(rs2740574)*	G/A	Substance specific function
*CYP3A5*	rs776746 c.219-237A > G (in *3)	G/G	No function (PM ^3^)
*ABCB1*	rs2032583 c.2685+49T > C *(rs1045642*, *rs1128503, rs2032582)*	T/T (WT ^4^)	Substance specific function

^1^ NM: normal metabolizer; ^2^ IM: intermediate metabolizer; ^3^ PM: poor metabolizer; ^4^ WT: wild type.

**Table 3:** Case #2 medication at the time of the reported severe ADRs.

Substance	Schedule
Quetiapine XR ^1^ 200 mg	0-0-1-0
Quetiapine 100 mg	0-0-0-1
Escitalopram 10 mg	1-0-0-0
Metoprolol DR ^2^ 25 mg	1-0-0-0
Lisinopril 10 mg	1-0-0-0

^1^ XR: extended release; ^2^ DR: delayed release.

**Table 4:** Case #2 selected results of the panel-pharmacogenotyping and phenotype interpretation.

Gene	Variant *(Also Tested Variants in Gene Locus)*	Genotype	Predicted Phenotype
*CYP1A2*	rs762551 g.75041917C > A (in *1F) *(rs2069514)*	C/A	Increased inducibility
*CYP2B6*	*(rs8192709*, *rs28399499*, *rs3745274)*	WT ^4^, *1	Normal function (NM ^1^)
*CYP2C19*	rs4244285 c.681G > A (in *2) *(rs4986893*, *rs12248560*, *rs28399504)*	G/A	Decreased function (IM ^2^)
*CYP2D6*	rs3892097 c.506-1G > A (in *4)	G/A C/T	Decreased function (IM ^2^)
	rs1065852 c.100C > T (in *4) *(CNV*, *rs35742686*, *rs5030655*, *rs5030867*, *rs5030865*, *rs5030656*, *rs201377835*, *rs28371706*, *rs59421388*, *rs28371725)*		
*CYP3A4*	*(rs2242480*, *rs2740574)*	WT ^4^, *1	Substance-specific function
*CYP3A5*	rs776746 c.219-237A > G (in *3)	G/G	No function (PM ^3^)
*ABCB1*	rs2032583 c.2685+49T > C *(rs1045642*, *rs1128503*, *rs2032582)*	T/T	Substance-specific function

^1^ NM: normal metabolizer; ^2^ IM: intermediate metabolizer; ^3^ PM: poor metabolizer; ^4^ WT: wild type.

## Data Availability

The data presented in this study are available on request from the corresponding author. The data are not publicly available for ethical and privacy reasons.
